# Supplementary material for: Factors influencing maternal death in Cambodia, Laos, Myanmar, and Vietnam countries: A systematic review
Source: PLoS One. 2024 May 17;19(5):e0293197. doi: 10.1371/journal.pone.0293197 (PMC11101123; doi:10.1371/journal.pone.0293197)
Supplement: S1 Checklist — (DOCX) [file pone.0293197.s001.docx]

**PRISMA 2020 CHECKLIST**

**Factors influencing maternal death in Cambodia, Laos, Myanmar, and Vietnam (CLMV) countries:**

**A systematic review**

| **Section and Topic** | **Item #** | **Checklist item** | **Location where item is reported** |
| --- | --- | --- | --- |
| **TITLE** | | |  |
| Title | 1 | Identify the report as a systematic review. | Under the sub-heading "Research Design and Question" of "Material and Method" (Lines -174) |
| **ABSTRACT** | | |  |
| Abstract | 2 | See the PRISMA 2020 for Abstracts checklist. | Mentioned in the section "Abstract". (Lines -25-57) |
| **INTRODUCTION** | | |  |
| Rationale | 3 | Describe the rationale for the review in the context of existing knowledge. | Under the heading "Introduction"  (Lines- 152-160) |
| Objectives | 4 | Provide an explicit statement of the objective(s) or question(s) the review addresses. | Under the heading "Introduction"  (Lines- 161-165) |
| **METHODS** | | |  |
| Eligibility criteria | 5 | Specify the inclusion and exclusion criteria for the review and how studies were grouped for the syntheses. | Under the sub-headings " "Inclusion and Exclusion Criteria” of "Material and Method” and Table 1.  (Lines – 188-227) |
| Information sources | 6 | Specify all databases, registers, websites, organizations, reference lists and other sources searched or consulted to identify studies. Specify the date when each source was last searched or consulted. | Under the sub-heading "Strategic paper search and selection” of "Material and Method" and Table 2.  (Lines 229-256) |
| Search strategy | 7 | Present the full search strategies for all databases, registers and websites, including any filters and limits used. | Under the sub-heading " Strategic paper search and selection” of "Material and Method" and Table 4. |
| Selection process | 8 | Specify the methods used to decide whether a study met the inclusion criteria of the review, including how many reviewers screened each record and each report retrieved, whether they worked independently, and if applicable, details of automation tools used in the process. | Under the sub-heading " Strategic paper search and selection” of "Material and Method" and Figure 3.  (Lines – 300-305) |
| Data collection process | 9 | Specify the methods used to collect data from reports, including how many reviewers collected data from each report, whether they worked independently, any processes for obtaining or confirming data from study investigators, and if applicable, details of automation tools used in the process. | Under the sub-heading "Data Extraction Template” of "Material and Method".  (Lines 307-314). |
| Data items | 10a | List and define all outcomes for which data were sought. Specify whether all results that were compatible with each outcome domain in each study were sought (e.g. for all measures, time points, analyses), and if not, the methods used to decide which results to collect. | The operational definitions of the variables included in the data extraction template were specified in Table 3 under the heading" "Material and Method"  Under the sub-headings " Strategic paper search and selection” of "Material and Method"  (Line 248-256) |
|  | 10b | List and define all other variables for which data were sought (e.g. participant and intervention characteristics, funding sources). Describe any assumptions made about any missing or unclear information. | The operational definitions of the variables included in the data extraction template were specified in Table 3 under the heading" "Material and Method"  Under the sub-headings " Strategic paper search and selection "Material and Method"  (Lines 248-256) |
| Study risk of bias assessment | 11 | Specify the methods used to assess the risk of bias in the included studies, including details of the tool(s) used, how many reviewers assessed each study and whether they worked independently, and if applicable, details of automation tools used in the process. | Under the sub-heading "Quality Appraisal Tool" of "Material and Method"  (Lines 315-327) |
| Effect measures | 12 | Specify for each outcome the effect measure(s) (e.g. risk ratio, mean difference) used in the synthesis or presentation of results. | The operational definitions of the variables included in the data extraction template were specified under the heading" "Material and Method"  Under the sub-headings " Strategic paper search and selection” of "Material and Method"  (Lines 269-243)  (Lines 329-341) |
| Synthesis methods | 13a | Describe the processes used to decide which studies were eligible for each synthesis (e.g. tabulating the study intervention characteristics and comparing against the planned groups for each synthesis (item #5)). | Under the sub-heading "Data Extraction and Analysis" of "Material and Method"  (Lines 329-341) |
|  | 13b | Describe any methods required to prepare the data for presentation or synthesis, such as handling of missing summary statistics, or data conversions. | Under the sub-heading "Data Extraction and Analysis" of "Material and Method"  (Lines 329-341) |
|  | 13c | Describe any methods used to tabulate or visually display results of individual studies and syntheses. | Under the sub-heading "Data Extraction and Analysis" of "Material and Method"  (Lines 329-341) |
|  | 13d | Describe any methods used to synthesize results and provide a rationale for the choice(s). If meta-analysis was performed, describe the model(s), method(s) to identify the presence and extent of statistical heterogeneity, and software package(s) used. | Under the sub-heading "Data Extraction and Analysis" of "Material and Method"  (Lines 329-341) |
|  | 13e | Describe any methods used to explore possible causes of heterogeneity among study results (e.g. subgroup analysis, meta-regression). | Under the sub-heading "Data Extraction and Analysis" of "Material and Method"  (Lines 329-341) |
|  | 13f | Describe any sensitivity analyses conducted to assess robustness of the synthesized results. | Not applicable |
| Reporting bias assessment | 14 | Describe any methods used to assess risk of bias due to missing results in a synthesis (arising from reporting biases). | Under the sub-headings "Quality Appraisal Tool" of "Material and Method" (Lines 315-327) |
| Certainty assessment | 15 | Describe any methods used to assess certainty (or confidence) in the body of evidence for an outcome. | Not applicable |
| **RESULTS** | | |  |
| Study selection | 16a | Describe the results of the search and selection process, from the number of records identified in the search to the number of studies included in the review, ideally using a flow diagram. | Under the sub-heading "Paper Selection Process Summary" of "Results"  (Lines 353-361) |
|  | 16b | Cite studies that might appear to meet the inclusion criteria, but which were excluded, and explain why they were excluded. | Under the sub-heading " Paper Selection Process Summary " of "Results"  (Lines 353-361) |
| Study characteristics | 17 | Cite each included study and present its characteristics. | Under the sub-heading "Background information of selected studies" of "Results" (Lines 365-388) |
| Risk of bias in studies | 18 | Present assessments of risk of bias for each included study. | Under the sub-heading "Background information of included studies" and in Table 5.  (Lines 383-388) |
| Results of individual studies | 19 | For all outcomes, present, for each study: (a) summary statistics for each group (where appropriate) and (b) an effect estimate and its precision (e.g. confidence/credible interval), ideally using structured tables or plots. | Under the sub-heading "Factors influencing contraceptive compliance" of "Results" and in Tables 6, 7, and 8.  (Lines 390-531. |
| Results of syntheses | 20a | For each synthesis, briefly summarise the characteristics and risk of bias among contributing studies. | Under the sub-heading "Factors influencing contraceptive compliance" of "Results" and in Tables 6, 7, and 8.  (Lines 390-531. |
|  | 20b | Present results of all statistical syntheses conducted. If meta-analysis was done, present for each the summary estimate and its precision (e.g. confidence/credible interval) and measures of statistical heterogeneity. If comparing groups, describe the direction of the effect. | Under the sub-heading "Factors influencing contraceptive compliance" of "Results" and in Tables 6, 7, and 8.  (Lines 390-531. |
|  | 20c | Present results of all investigations of possible causes of heterogeneity among study results. | Under the sub-heading "Factors influencing contraceptive compliance" of "Results" and in Tables 6, 7, and 8.  (Lines 390-531. |
|  | 20d | Present results of all sensitivity analyses conducted to assess the robustness of the synthesized results. | Under the sub-heading "Factors influencing contraceptive compliance" of "Results" and in Tables 6, 7, and 8.  (Lines 390-531. |
| Reporting biases | 21 | Present assessments of risk of bias due to missing results (arising from reporting biases) for each synthesis assessed. | Under the sub-heading "Background information of included studies" and in Table 5.  (Lines 383-388) |
| Certainty of evidence | 22 | Present assessments of certainty (or confidence) in the body of evidence for each outcome assessed. | Not Applicable |
| **DISCUSSION** | | |  |
| Discussion | 23a | Provide a general interpretation of the results in the context of other evidence. | Under the heading "Discussion"  (Lines 540-766) |
|  | 23b | Discuss any limitations of the evidence included in the review. | Under the sub-heading "Limitations" in the heading "Conclusion"  (Lines 788-794) |
|  | 23c | Discuss any limitations of the review processes used. | Under the sub-heading "Limitations" in the heading "Conclusion"  (Lines 788-794) |
|  | 23d | Discuss implications of the results for practice, policy, and future research. | Under the heading " Dissemination plan" (Lines 802-813) |
| **OTHER INFORMATION** | | |  |
| Registration and protocol | 24a | Provide registration information for the review, including register name and registration number, or state that the review was not registered. | Under the sub-heading "Ethical consideration" in the heading "Material and Method"  (Lines 343-350) |
|  | 24b | Indicate where the review protocol can be accessed, or state that a protocol was not prepared. | Under the sub-heading "Ethical consideration" in the heading "Material and Method" (Lines 343-350) |
|  | 24c | Describe and explain any amendments to the information provided at registration or in the protocol. | Under the sub-heading "Ethical consideration" in the heading "Material and Method"(Lines 343-350) |
| Support | 25 | Describe sources of financial or non-financial support for the review, and the role of the funders or sponsors in the review. | Under the heading "Administrative information and author contributions"  Lines 809-825. |
| Competing interests | 26 | Declare any competing interests of review authors. | Under the heading "Administrative information and author contributions"  Lines 809-825. |
| Availability of data, code and other materials | 27 | Report which of the following are publicly available and where they can be found: template data collection forms; data extracted from included studies; data used for all analyses; analytic code; any other materials used in the review. | Under the heading "Access to data and supporting information”  (Lines 796-840) |
