# Supplementary material for: Factors influencing maternal death in Cambodia, Laos, Myanmar, and Vietnam countries: A systematic review
Source: PLoS One. 2024 May 17;19(5):e0293197. doi: 10.1371/journal.pone.0293197 (PMC11101123; doi:10.1371/journal.pone.0293197)
Supplement: Attachment — Submitted filename: Response to Reviewers.doc [file pone.0293197.s003.doc]

**Response to Reviewers**

| Number | Original comments of the reviewer | Reply by the author(s) | Changes done on the page number and line number |
| --- | --- | --- | --- |
| 10 January 2023 | | | |
| 1 | Please update the methods section to specify the date when each source was last searched or consulted, as requested by the PRISMA checklist. | As suggested by the Journal editor, the authors mentioned the start and end dates of the literature search under the title "*Organizing review team of Search method"* in the *Material and method section* as follows,  " The independent literature search was started in Hinari, Google Scholar and PubMed on 13 July 2022, 28 July 2022 and 11 August 2022 and stopped in all databases on 1^st^ September 2022. | Line numbers 261-263. |
