# Supplementary material for: Factors influencing maternal death in Cambodia, Laos, Myanmar, and Vietnam countries: A systematic review
Source: PLoS One. 2024 May 17;19(5):e0293197. doi: 10.1371/journal.pone.0293197 (PMC11101123; doi:10.1371/journal.pone.0293197)
Supplement: Attachment — Submitted filename: feedback PONE maternal death systematic review .docx [file pone.0293197.s004.docx]

**Factors influencing maternal death in Cambodia, Laos, Myanmar, and Vietnam (CLMV) countries: A systematic review**

**Summary of the Research**

A maternal mortality ratio is a sensitive indicator when comparing the overall maternal health between countries. This very high figure could be assumed to be the failure of maternal healthcare efforts. The CLMV (Cambodia, Laos, Myanmar, and Vietnam) countries are the low-income countries of the South-East Asia region where their maternal mortality ratios are disproportionately high. This systematic review aimed to summarize all possible factors influencing maternal mortality in CLMV countries. This systematic review applied "The Preferred Reporting Items for Systematic reviews and Meta-Analyses (PRISMA) Checklist (2020)", for the report frame, "PICOT" criteria for the review question and a simple random sampling procedure for choosing three academic databases randomly. For critical work,  JBI critical appraisal tool sets were chosen. The data analysis included two stages: descriptive analysis of the general information of the included papers and qualitative analysis of key  findings. This review concluded that poor family income, illiteracy, low education levels and agricultural and unskilled manual job types contributed to insufficient antenatal care. Non-marital status and sex-associated work were highly associated with induced abortions. Being rural women, certain social and cultural beliefs and husbands' influences directly contributed to the limitations of maternal healthcare services. Early and elderly age at marriage, over 35 years, multigravida and higher parity were more likely to contribute to poor maternal healthcare outcomes. Higher unmet needs for maternal healthcare services occurred among women living far from healthcare facilities. Regarding the maternal healthcare workforce, the quality and number of healthcare providers, the development of healthcare infrastructures and human resource management policy appeared to be arguable. Concerning maternal healthcare service use, the provisions of mobile and outreach maternal healthcare services were convenient and limited. Low utilization rates were due to several supply-side constraints.

**Financial Disclosure:** in this section, please write what are the requirement; for example, describes the sources of funding for your study.

**Describe where the data may be found in full sentences. If you are copying our sample text, replace any instances of XXX with the appropriate details:** in this section, please provide detail information about the papers according to the reequipment in this section and the reasons for selecting unpublished papers. As I understand from your systematic review, papers for review were published and you used JBI. Please review this section.

**Areas for improvement**

**Title:**

The authors wrote the title in a good way reflecting the aim of the systematic review. However, the author can select another short title, a bit different from the main title. For the title **Factors influencing maternal death in Cambodia, Laos, Myanmar, and Vietnam (CLMV) countries: A systematic review:** use capital each word and removed abbreviation (CLMV).

**Abstract:**

The authors should make sure that they use standard abbreviations in the manuscript. They should keep the abbreviations to a minimum.

The authors should revise the language to improve readability.

In results section please rewrite clearly the result of the review.

The authors should mention clearly the main aim and objective of the systematic review.

In the keywords section of the abstract you mentioned Keywords: Influencing Factors, maternal Mortality and Health Outcome, and CLMV  Countries: no need to mention influencing (factors are enough), you can put maternal one key word and mortality another key word. Also no need to mention the countries in the keywords.

The authors should make sure that the abstract don’t exceed 300 words. The abstract almost 400 words in your review.

**Introduction Section:**

**In the first paragraph,**

The authors should mention the reference of maternal death.

A maternal mortality ratio does not concern maternal death from accidental or incidental causes: please continue the sentence (what maternal mortality concern of?)

In 2020, for every 100,000 LB: what is LB, do you mean labour, Please write in a full form.

In this sentence (preventable maternal causes): explain what do you mean by preventable maternal causes and provide example please.

Many countries around the world calculate the maternal mortality ratio to determine their individual and social development: reference is needed here.

The authors should revise the first paragraph for grammar issues and language to improve readability.

**In the second paragraph,**

For many years, the World Health Organization (WHO) has been exploring ways to overcome the inequalities in maternal mortality between low-income and high-income countries: reference is needed here.

In these ways, provision of nutrition supplementation, promotion of maternal immunization through outreach strategies, training volunteer health workers (Axillary Mid-wives, Traditional Birth Attendants and Community Health Workers), training healthcare providers, upgrading maternal healthcare infrastructure and equipment, promoting accessibility to maternal healthcare services, and promoting the awareness were included: too long sentence, please make it short and more readable.

Moreover, in 2001, eight goals of the Millennium Development Goals (MDGs) were set and implemented to reduce the maternal mortality ratios in 1990 by three quarters between 2000 and 2015: rewrite the sentence to make it more readable and put a reference at the end of the sentence.

Because of these interventions, the global maternal mortality ratio (MMR) was reduced by 45%, MMR in Southeast Asia was reduced by 64%, the global skill birth attendance rate was increased by 12%, global quality antenatal coverage was increased by 39%, and global contraceptive prevalence among married women was increased by 9% between 2000 and 2015: long sentence, please try to divided it to many sentences. Put the reference. Also, it is not clear is this interventions a protocol in southeast Asia or this is a study. Try to clarify. Mention which countries these intervention implemented in Southeast Asia.

However, after analysis of MMR at the end of 2015, MMR was reduced by less than half of the MDG targeted settings and more effective interventions still need to reach the predetermined MDG goals among LMICs including CLMV countries: please mention what is LMICs, you mentioned that in the next sentence.

In many low- and middle-income countries (LMICs), responsibility for changing the constant or slow declination of maternal mortality trends into the targeted line of the health-related Millennium Development Goals (MDGs) 5 remained a major challenge: rewrite the sentence, not clear. The authors should revise the language to improve readability. Also, reference is needed here.

In the goal of MDG 5, the first and second targets were "reducing the maternal mortality ratio by three-quarters" and "achieving universal access to reproductive healthcare services" at the end of the 25 years duration after 1990: reference is needed here.

**In the third paragraph,**

Consequently, world leaders adopted the 2030 Agenda including the 17 sustainable development goals by replacing eight MDGs to end all preventable causes of maternal deaths: who are world leaders, what is 2030 agenda, rewrite and clarify this sentence, reference is needed.

As announced in the SDG context, countries have rallied along with a new goal to drive the action of reduction in maternal mortality by 2030 as well as to ensure that by 2030, global MMR is to be less than 70/100,000 LB and MMR of each country is not to be more than twice  the average of global MMR: what is SDG, you mentioned before MDGs. Rewrite the sentence to make more readable please.

The CLMV (Cambodia, Laos, Myanmar, and Vietnam) countries are the low-income countries of the South-East Asia region where their maternal mortality ratios are disproportionately high: please write the reference. No need to mention the countries you did before.

Through the national commitments of the health-related sustainable development goals (SDGs), CLMV countries are also responsible to reduce their maternal mortality ratios to < 70/100,000 LB (Live Births) by 2030: reference is needed here. Please rewrite this sentence to make it more readable.

**In the fourth paragraph,**

Maternal Death Surveillance and Response (MDSR) is recognized as another option for maternal death-reducing intervention as well as this concept is widely established globally as a sufficient intervention in the era of MDGs: reference is needed here.

When surveying the MDSR implementation of the LMICs in 2015, the survey results showed that the notification and reviewing rate of all maternal deaths was 86% and 85%, 24% of LMICs had no MDSR review committee, and 54% of LMICs could not review MDSR at least biannually: please rewrite this sentence to make it more readable and provide a reference at the end of the sentence.

**In the fifth paragraph,**

Historically, the CLMV countries have been colonized by Western powers: provide example of Western powers. The Authors need to decide if this sentence is important to be added.

Myanmar was part of British India from 1824 to 1948, and Cambodia Laos and Vietnam were part of French Indochina from 1887 to 1954: reference is needed here.

In 2019, the population of CLMV countries was 174 million and this is approximately 25% of the population of Association of South East Asia Nations (ASEAN) members and 3.6% of the total population of Asia: there is no connection between this sentence and the previous one. provide a reference here.

Gross domestic product (GDP)  per capita in 2019 averaged 2,200 US$, much lower than the ASEAN average of 12,979 US$ and the Asian average of 17,909 US$: The authors should remove the abbreviation GDP as it is not well known or international. The authors also should think about the necessary of this sentence. Also to make sure that all sentences are well connected to each other.

The CLMV countries managed to  reduce the maternal mortality rate by 32% to reach the target of a 2/3 reduction in MMR over years (from 1990 to 2015): reference is needed here.

In the CLMV countries, the average MMR of Cambodia from  1990 to 2020 was 276/100,000 LB and the measure in 2020 indicates that 206/100,000 LB need to be reduced for achieving the SDG target by 2030 (<70/100,000 LB). The average MMR of Lao PDR from 1990 to 2020 was 362/100,000 LB and the 2020 MMR data indicates that 292/100,000 LB need to be dropped between 2021 and 2030. The average MMR of Myanmar from 1990 to 2020 was 186 and the 2020 MMR data pointed out that it needs to drop more than 116/100,000 LB to meet the SDG target (less than 70/100,000 LB). When looking at Vietnam, the average MMR from 1990 to 2020 was 199/100,000 LB: references are needed here. Make good connection to these sentences to make them more readable.

The maternal mortality trends among CLMV countries (1990-2020) were shown in S2 Fig: write page number of S2, so the readers can access fast and easily.

**In the sixth paragraph,**

When estimating the average annual MMR (per 100,000 LB) of reduction, 7.1 could be reduced in Cambodia, 14.9  in Laos, 0.4 in Myanmar and 3 in Vietnam: please write the reference here.

In these cases, among CLMV  countries, there are still huge differences between the average annual MMR of reduction and the annual MMR needed to achieve the SDG target: reference is needed here.

**Overall**

The authors need to arrange introduction section. Rewrite some sentences so, the readers will understand what you wanted to convey from messages. The authors should make sure that missing references are written within the introduction section. They also need to make sure to clearly mention the significant of the systematic review and the aim of it. The authors should indicate what they wanted the readers to understand. The authors should indicate the significance of the systematic review and the overall aim of this systematic review.

**Material and Methods:**

**Research Design,**

The authors should write one sentence on how to limit the bias for this systematic review.

(available at <https://prisma.shinyapps.io/checklist/>): no need to mention this in the paper, however, the authors can mention or refer this link in the appendix.

The research question of the review was "Which factors are influencing the improvement of maternal deaths in Cambodia, Laos, Myanmar, and Vietnam, CLMV countries?": please, rewrite the question in a better way. For example, what are the factors affecting……?

(see in S2 Fig): the authors should mention the page number, so the reader can access to information better and fast.

According to the argumentation of a study, the appropriateness and specificity …..: Delete according to the argumentation.

In Table 1. Research question and FINER criteria: in Feasible (F), why did you exclude purchased papers or studies? Provide reasons. in Interesting (I), you wrote (Found more effective ways to reduce MMR to the referenced targets of the SDG goals) but you aim is to know the factor, please check that and if you have more than one aim, please write them.

The authors should mention if a protocol is exist for their systematic review.

**Inclusion/ exclusion criteria,**

The elements of PICOT that were mentioned in S3 Fig addressed the inclusion criteria of this review: please write page number for S3.

The authors should write clearly PICOT stand for what, so the readers will understand.

The authors should separate the paragraphs of inclusion criteria and exclusion criteria, so the reader will follow the systemic review in more clear way.

This review excluded secondary analysis and evidence-based information that has previously been published as historical documents, systematic reviews or/and meta-analyses, reports, books, handouts, posters, factsheets, encyclopedias, student thesis, and conference proceedings: The authors should provide reasons for exclusion briefly and what the authors means when they wrote exclude secondary analysis that has previously been published.

"PICOT" framework (S2 Fig). Please write the page number of S2 figure.

**Literature Search,**

No comments.

**Selection of Information Sources,**

…s, etc.,: please, no need the write extra. Just write what you searched. Or delete this sentence since you used electronic resources only.

Table 2. List of the Selected Academic Research Databases and their Descriptions: the authors can put this table in appendix and then refer it with page number.

The authors can write the number of study from these three database briefly.

**Defining Key Search Terms,**

"Influencing Factors", "Maternal Mortality and Health Outcome": I think these key words should be separated, for example no need to mention influencing factors (factor is enough), maternal is one key word and mortality is another key word. Please check that.

**Search Method,**

**Organizing review team,**

coordinator of STIMU: please, write the full form of STIMU.

on 13 July 2022, 28 July 2022 and 11 August 2022 and stopped in all databases on 1st September 2022: I think you need to mention the date from …to…. For example, for 13 July 2022 to 1^st^ Sep 2022.

**Developing Tools,**

The researcher: The authors have to be consistent using the word the researcher or the researchers or investigators in all document.

The authors should check grammar for readability.

**Evaluation of Search Results,**

No comments.

**Study Selection,**

Details of selection processes were explained in the finding section: please refer to the page number, so the readers can get access fast.

PRISMA: the authors can provide diagram of the PRISMA in this systematic review.

**Information Extraction,**

DET: the authors have to write the full form of this abbreviation.

**Quality Appraisal,**

The authors should provide the reason for selecting JBI.

The authors should check grammar for readability.

**Data Analysis,**

In qualitative analysis, among different approaches: content analysis, narrative analysis, discourse analysis, framework analysis and grounded theory, this review analyzed all key extracted information through content analysis: the authors should provide reasons for selecting these data analysis. It is well known in grounded theory that constant comparative analysis.

Finally, the highlighted major themes were summarized and assembled according to the representatives of eight themes: Please write these 8 themes.

**Ethical Consideration,**

STIMU and SSCHREC: Please write the full form of these two abbreviations.

Overall, The authors should revise the language and grammar to improve readability. I think, the authors should arrange this section in a better way. Please refer to the guideline of this journal.

**Results:**

**Summary Of Paper Selection Process,**

The authors should move this section to the PRISMA.

Two investigators first identified 1112 titles and abstracts by searching PubMed (31.3%), Google Scholar (44.6%), and Hinari (24.1%) records: the authors should mention the number of studies identified in each database, I don’t understand what is the meaning of the percentages of each database.

Inter-reviewer agreement form: the authors should explain the meaning of this form briefly. Then, the authors should mention how many papers excluded and why.

see in S1 Fig: mention the page number please after fig.

**Background Information of Included Studies,**

Since this study concentrated on the CLMV countries, all included studies took place in these four countries. 27 (36%) of the 75 studies were from Myanmar, 23 (31%) from Laos, 13 (17%) from Cambodia, and 12 (16%) from Vietnam: I don’t think there is a need to mention percentages after each number, check that please for all document.

In this review, 31 (41.3%) of the papers were published between 2019 and 2022, 19 (25.3%)  between 2010 and 2014, and 25 (33.33%) between 2015 and 2018: the authors should arrange papers according to the years, 2015 before 2014.

The quantitative information in this review was gathered using semi-structured interview questionnaires (83%) and the qualitative information was gathered through focus group discussions and key informant interviews (17%): the authors need to mention number of papers. Be consistence in all document, I think number if papers is better than percentage.

According to the JBI criteria, some cross-sectional studies (12.5%, n=6) did not mention pre- testing data collection instruments and Cronbach alpha values as well as the standardization of the instruments. And (25%, n=12) studies also did not explain how they identified the confounding factors: did these affected the review in some way, please explain briefly.

**Factors Influencing Maternal Death,**

**Socio-Economic Factors,**

The authors should join some paragraphs together which talk about the same concept.

In terms of male involvement, having a supportive husband was positively correlated with current contraceptive use, antenatal care practices, and delivery plans. Women who had  husbands who supported contraception were more likely to succeed in family planning than  other women [29]. Similarly, the studies concluded that favorable decisions made by husbands, gender, and societal norms had a significant impact on women's access to antenatal care, contraceptive choice, and delivery options [30, 31]. However, it was discovered that most husbands' perspectives were that discussions about pregnancy care, family planning, and delivery cases were not their concerns [32]: The author should check if husband support will be under the concept of socio-economic factors.

The authors should check that all the references in Table 6. The influence of socioeconomic factors on accessing maternal health care services and high risk of maternal deaths are correlated with the text.

**Maternal Biological Factors,**

Early and older marriage ages were found to increase the likelihood of not receiving quality antenatal care, and elderly pregnant women were more likely to rely on traditional therapies. Early childbirth age was statistically associated with birth-related complications and missed postnatal follow-up care (Table 7) [22, 34]: the authors should mention if marriage age affect MMR within these studies.

**Maternal Health Awareness-Associated Factors,**

According to one study, approximately one-third of HIV-positive pregnant women had less access to maternal health education and family planning education [48]: please mention the result of this study if MMR increased.

Furthermore, young women and primigravidae had significantly lower rates of sexual reproductive health discussion [47] and rural mothers had a lower awareness of pregnancy and childbirth complications, as well as postpartum dietary restrictions [49, 50]: again, the authors need to discuss if the concept of these studies like health education or awareness had affect MMR negatively or positively.

**Maternal Health Need-Associated Factor,**

The authors need to separate the table of this factor from the next factor.

**Maternal Psychological Factors,**

The authors need to separate the table of this factor from the previous factor.

**The Status and Quality of The Maternal Healthcare Workforce,**

It was also claimed that TBA handled pregnancy and childbirth complications in an unscientific and unsafe manner, which could result in maternal deaths [18]: The authors should clarify and mention what is the meaning of TBA abbreviation. ABA Stand for what?

The authors should revise the grammar for more readability.

Furthermore, underdevelopment of healthcare infrastructures and a lack of medical supplies were identified as major barriers to providing effective care and high quality services [21, 62]: I think this should come under the next factor as it discuss the services.

**Maternal Healthcare Services Use-Associated Factors,**

The authors should revise the grammar for more readability.

**Community-Associated Factors,**

The authors need to discuss how the community associated factors affected MMR.

The authors should revise the grammar for more readability.

**Discussion:**

In this study, family income was shown to be a strong and significant factor that was substantially related to maternal and reproductive healthcare access and maternal mortality: mention review instead of study and mention the references discuss this concept.

The authors discussed the all factors but need to discuss from different angles. They should discuss clearly about the issues found from different angels.

The authors should discuss each themes from different angle and in detail.

The authors should revise the language and grammar to improve readability.

Most of discussion section was recommendation, the authors should revised this section.

The authors should discuss the results in consistent with the previous section.

Overall, Discussion section should be revised totally and should be discussed from different angels

**Conclusion,**

No comments

**Limitation and remaining sections,**

No comments

**References:**

The authors should revise all references according to the guidelines provided.
