# Supplementary material for: Factors influencing maternal death in Cambodia, Laos, Myanmar, and Vietnam countries: A systematic review
Source: PLoS One. 2024 May 17;19(5):e0293197. doi: 10.1371/journal.pone.0293197 (PMC11101123; doi:10.1371/journal.pone.0293197)
Supplement: Attachment — Submitted filename: Response to Reviewers (5.10.2023).doc [file pone.0293197.s006.doc]

**Response to Reviewers**

| Number | Original comments of the reviewer | Reply by the author(s) | Changes done on the page number and line number |
| --- | --- | --- | --- |
| 10 January 2023 | | | |
| 1 | Please update the methods section to specify the date when each source was last searched or consulted, as requested by the PRISMA checklist. | As suggested by the Journal editor, the authors mentioned the start and end dates of the literature search under the title "*Organizing review team of Search method"* in the *Material and method section* as follows,  " The independent literature search was started in Hinari, Google Scholar and PubMed on 13 July 2022, 28 July 2022 and 11 August 2022 and stopped in all databases on 1^st^ September 2022. | Line numbers 261-263. |
| August 2, 2023 | | | |
| 1 | **Financial Disclosure:** in this section, please write the requirements; for example, describe the funding sources for your study. | ***Financial disclosure:*** This research is part of a student dissertation and was completed by the researcher's own fund. | Line numbers -59-60. |
| 2 | The authors wrote the title in a good way reflecting the aim of the systematic review. However, the author can select another short title, a bit different from the main title. For the title **Factors influencing maternal death in Cambodia, Laos, Myanmar, and Vietnam (CLMV) countries: A systematic review:** use capital each word and removed abbreviation (CLMV). | The authors corrected the title: " **Factors Influencing Maternal Death in Cambodia, Laos, Myanmar, and Vietnam Countries: A Systematic Review:** | Line number -1-3. |
| 3 | The authors should make sure that they use standard abbreviations in the manuscript. They should keep the abbreviations to a minimum. The authors should revise the language to improve readability. | The authors removed some abbreviations and improved the language." "JBI", | Line number 41. |
| 4 | In results section please rewrite clearly the result of the review. | **The authors rewrote the results.**  **" *Results*:** Poor family income, illiteracy, low education levels, living in poor households, and agricultural and unskilled manual job types of mothers contributed to insufficient antenatal care. Maternal factors like non-marital status and sex-associated work were highly associated with induced abortions while being rural women, ethnic minorities, poor maternal knowledge and attitudes, certain social and cultural beliefs and husbands' influences directly contributed to the limitations of maternal healthcare services. Maternal factors that made more contributions to poor maternal healthcare outcomes included lower quintiles of wealth index, maternal smoking and drinking behaviours, early and elderly age at marriage, over 35 years pregnancies, unfavourable birth history, gender-based violence experiences, multigravida and higher parity . Higher unmet needs and lower demands for maternal healthcare services occurred among women living far from healthcare facilities. Regarding the maternal healthcare workforce, the quality and number of healthcare providers, the development of healthcare infrastructures and human resource management policy appeared to be arguable. Concerning maternal healthcare service use, the provisions of mobile and outreach maternal healthcare services were inconvenient and limited. Low utilization rates were due to several supply-side constraints." | Line numbers -44-60. |
| 5 | The authors should mention clearly the main aim and objective of the systematic review. | **The authors have already the main aim of the study such as"** This systematic review aimed to summarize all possible factors influencing maternal mortality in CLMV countries." | Line numbers – 32-33. |
| 6 | In the keywords section of the abstract you mentioned Keywords: Influencing Factors, maternal Mortality and Health Outcome, and CLMV Countries: no need to mention influencing (factors are enough), you can put maternal one key word and mortality another key word. Also no need to mention the countries in the keywords. | **As suggested by the reviewers, the authors corrected the keywords such as " *Keywords*:** Factors, Maternal Healthcare, Maternal Mortality and Health Outcome. | Line numbers- 64-65. |
| 7 | The authors should make sure that the abstract don’t exceed 300 words. The abstract almost 400 words in your review. | **The authors reduced the word count of abstracts to 344.** | Line numbers- 27-63. |
| Introduction Section  In the first paragraph | | | |
| 8 | The authors should mention the reference of maternal death.  A maternal mortality ratio does not concern maternal death from accidental or incidental causes: please continue the sentence (what maternal mortality concern of?) | **The author summarized these sentences like '**Maternal death is a woman's death and it is directly associated with maternal causes of pregnancy, childbirth and puerperium between the 1^st^ day of initiation of pregnancy and the 42^nd^ day of termination of pregnancy, but does not concern maternal death from accidental or incidental causes [1].'  **The citation number [1] was for these sentences.** | Line numbers – 70-73. |
| 9 | In 2020, for every 100,000 LB: what is LB, do you mean labour, Please write in a full form. | **The authors corrected LB "Live Births (LB).** | Line number- 73 |
| 10 | In this sentence (preventable maternal causes): explain what do you mean by preventable maternal causes and provide example please. | **The authors explained this sentence with information such as "**152 mothers died globally and 147 mothers also passed away in South Asia Region due to preventable causes of maternal deaths such as disparities in geographical and socioeconomic status, marital, pregnancy and childbirth-related age, and contraception [2]." | Line numbers -74-76. |
| 11 | Many countries around the world calculate the maternal mortality ratio to determine their individual and social development: reference is needed here. | **The authors cited these sentences with citation number [3].** | Line number- 83. |
| 12 | The authors should revise the first paragraph for grammar issues and language to improve readability. | **The authors revised the first paragraph such as "** Maternal death is a woman's death and it is directly associated with maternal causes of pregnancy, childbirth and puerperium between the 1^st^ day of initiation of pregnancy and the 42^nd^ day of termination of pregnancy, but does not concern other maternal death from accidental or incidental causes [1]. In 2020, for every 100,000 Live Births (LB), 152 mothers died globally and 147 mothers also passed away in South Asia Region due to preventable causes of maternal deaths such as disparities in geographical and socioeconomic status, marital, pregnancy and childbirth-related age, and contraception [2]. Many countries around the world calculate the maternal mortality ratio to determine their individual and social development. A maternal mortality ratio is a sensitive indicator when comparing the overall maternal health between the countries and its very high measure could be assumed as the failure of maternal healthcare efforts in a country. On the other hand, a low maternal mortality ratio indicates satisfaction with the overall status of women's health and the women's accessibility to reproductive healthcare services as well as reflects the responsiveness of the national health system to women's health needs [3]. | Line numbers – 70-83. |
| In the second paragraph | | | |
| 13 | For many years, the World Health Organization (WHO) has been exploring ways to overcome the inequalities in maternal mortality between low-income and high-income countries: reference is needed here. | **The citation number [3] has already been used for this information and this information is correlated with the following information.** | Line number -90 |
| 14 | In these ways, provision of nutrition supplementation, promotion of maternal immunization through outreach strategies, training volunteer health workers (Axillary Mid-wives, Traditional Birth Attendants and Community Health Workers), training healthcare providers, upgrading maternal healthcare infrastructure and equipment, promoting accessibility to maternal healthcare services, and promoting the awareness were included: too long sentence, please make it short and more readable. | **The authors rewrote this sentence such as "** In these ways, nutrition supplementation was provided and maternal immunization was promoted through outreach strategies. Besides, training volunteer health workers (Axillary Midwives, Traditional Birth Attendants and Community Health Workers), and healthcare providers, upgrading maternal healthcare infrastructure and equipment, promoting accessibility to maternal healthcare services, and awareness were also included [3]." | Line number – 85-91. |
| 15 | Moreover, in 2001, eight goals of the Millennium Development Goals (MDGs) were set and implemented to reduce the maternal mortality ratios in 1990 by three quarters between 2000 and 2015: rewrite the sentence to make it more readable and put a reference at the end of the sentence. | **The authors rewrote this sentence such as "** Moreover, in 2001, eight goals of the Millennium Development Goals (MDGs) were set and implemented to reduce the maternal mortality ratios [4]." | Line numbers -91-93. |
| 16 | Because of these interventions, the global maternal mortality ratio (MMR) was reduced by 45%, MMR in Southeast Asia was reduced by 64%, the global skill birth attendance rate was increased by 12%, global quality antenatal coverage was increased by 39%, and global contraceptive prevalence among married women was increased by 9% between 2000 and 2015: long sentence, please try to divided it to many sentences. Put the reference. Also, it is not clear is this interventions a protocol in southeast Asia or this is a study. Try to clarify. Mention which countries these intervention implemented in Southeast Asia. | **The reference number [4] is for this information.**  **The authors rewrote these sentences such as "** After adopting and implementing the WHO's maternal reduction strategies and global goals (MDGs-2001) among 189 countries, the global maternal mortality ratio (MMR) was reduced by 45%, MMR in Southeast Asia was reduced by 64%. Moreover, the global skill birth attendance rate was increased by 12%, global quality antenatal coverage was increased by 39%, and global contraceptive prevalence among married women was increased by 9% between 2000 and 2015.' | Line number - 102 |
| 17 | However, after analysis of MMR at the end of 2015, MMR was reduced by less than half of the MDG targeted settings and more effective interventions still need to reach the predetermined MDG goals among LMICs including CLMV countries: please mention what is LMICs, you mentioned that in the next sentence. | **The authors explained LMICs such as "Low- and Middle-income Countries".** | Line number -101. |
| 18 | In many low- and middle-income countries (LMICs), responsibility for changing the constant or slow declination of maternal mortality trends into the targeted line of the health-related Millennium Development Goals (MDGs) 5 remained a major challenge: rewrite the sentence, not clear. The authors should revise the language to improve readability. Also, reference is needed here.  In the goal of MDG 5, the first and second targets were "reducing the maternal mortality ratio by three-quarters" and "achieving universal access to reproductive healthcare services" at the end of the 25 years duration after 1990: reference is needed here. | **The reference number [5] is concerned with all information from line numbers 102 to 109.**  **"** In many LMICs, responsibility for changing the constant or slow declination of maternal mortality trends into the targeted line of the health-related Millennium Development Goals (MDGs) 5 remained a major challenge. In the goal of MDG 5, the first and second targets were "reducing the maternal mortality ratio by three-quarters" and "achieving universal access to reproductive healthcare services" at the end of the 25 years duration after 1990. Until 2017, many LMICs were under the level of the targeted achievement and there were nearly 800 pregnancy- and childbirth-associated deaths every day. More than 90% of global maternal deaths were contributed to LMICs [5]. | Line number – 102-109. |
| In the third paragraph | | | |
| 19 | Consequently, world leaders adopted the 2030 Agenda including the 17 sustainable development goals by replacing eight MDGs to end all preventable causes of maternal deaths: who are world leaders, what is 2030 agenda, rewrite and clarify this sentence, reference is needed. | **As recommended by the reviewers, the authors changed these sentences into "** Consequently, heads of states and governments from 189 countries adopted the 2030 Agenda launched by United Nations in September 2015 with the aims of ending all forms of poverty. The agenda included the 17 sustainable development goals and a goal was set to replace eight MDGs to end all preventable causes of maternal deaths. | Line number – 110-113. |
| 20 | As announced in the SDG context, countries have rallied along with a new goal to drive the action of reduction in maternal mortality by 2030 as well as to ensure that by 2030, global MMR is to be less than 70/100,000 LB and MMR of each country is not to be more than twice  the average of global MMR: what is SDG, you mentioned before MDGs. Rewrite the sentence to make more readable please. | **As recommended by the reviewers, the authors rewrote these sentences such as "** As announced by a United Nations Summit in the SDG context, countries have rallied along with a new goal to drive the action of reduction in maternal mortality by 2030. According to the agenda, global MMR is to be less than 70/100,000 LB and MMR of each country is not to be more than twice the average of global MMR at the end of 2030." | Line numbers- 114-118. |
| 21 | The CLMV (Cambodia, Laos, Myanmar, and Vietnam) countries are the low-income countries of the South-East Asia region where their maternal mortality ratios are disproportionately high: please write the reference. No need to mention the countries you did before. | **As recommended by the reviewers, the authors rewrote these sentences such as "**The CLMV countries are the low-income countries of the South-East Asia region where their maternal mortality ratios are disproportionately high [6]." | Line numbers- 118-120 |
| 22 | Through the national commitments of the health-related sustainable development goals (SDGs), CLMV countries are also responsible to reduce their maternal mortality ratios to < 70/100,000 LB (Live Births) by 2030: reference is needed here. Please rewrite this sentence to make it more readable. | **As recommended by the reviewers, the authors rewrote these sentences such as "** They are also responsible to reduce their maternal mortality ratios to < 70/100,000 LB (Live Births) by 2030 according to the SDGs of the United Nations Summit. In taking action the stakeholders of the CLMV countries perceived that they lacked ownership, some SDG goals were less relevant to the local development contexts and the strategies were more likely to be related to donors' wants. Therefore, for the CLMV countries, their particular national strategic plans gave the preferences to their local needs and have been prioritized. Through the energetic work of the technical working group, more specific plans based on their local needs are sustainably developed, their achievements in maternal health are regularly reviewed and their developed plans, policies and achievements are assessed for the alignments of SDGs [6]." | Line numbers- 120-130. |
| **In the fourth paragraph,** | | | |
| 23 | Maternal Death Surveillance and Response (MDSR) is recognized as another option for maternal death-reducing intervention as well as this concept is widely established globally as a sufficient intervention in the era of MDGs: reference is needed here. | The authors have already cited these sentences with the reference number [7] like "Maternal Death Surveillance and Response (MDSR) is recognized as another option for maternal death-reducing intervention as well as this concept is widely established globally as a sufficient intervention in the era of MDGs. It is a new approach in which causes of maternal deaths and the possible determinant factors are reviewed and the appropriate responses are undertaken according to the basis of the feedback and recommendation evolved from the MDSR establishment [7].'' | Line numbers 117-122 |
| 24 | When surveying the MDSR implementation of the LMICs in 2015, the survey results showed that the notification and reviewing rate of all maternal deaths was 86% and 85%, 24% of LMICs had no MDSR review committee, and 54% of LMICs could not review MDSR at least biannually: please rewrite this sentence to make it more readable and provide a reference at the end of the sentence. | **The author rewrote these sentences like "**According to the survey on the MDSR implementation of the LMICs in 2015, the record showed that, of LMICs, only 86% have notified the maternal death cases and 85% reviewed determinants of the reported maternal death cases. 24% of LMICs had no MDSR review committee, and 54% of LMICs could not review MDSR at least biannually.  The authors have already cited these sentences with the reference number [7]. | Line numbers- 127-130. |
| In the fifth paragraph | | | |
| 25 | Historically, the CLMV countries have been colonized by Western powers: provide an example of Western powers. The Authors need to decide if this sentence is important to be added. | As per the reviewer's suggestion, the authors corrected that " Historically, the CLMV countries have been explored, conquered, colonized and settled by many European countries, causing the CLMV countries less development for a long time." | Line numbers -133-134. |
| 26 | Myanmar was part of British India from 1824 to 1948, and Cambodia Laos and Vietnam were part of French Indochina from 1887 to 1954: reference is needed here. | The author changed and added reference to these sentences "In evidence, Myanmar was part of British India from 1824 to 1948, and Cambodia Laos and Vietnam were part of French Indochina from 1887 to 1954 [8]." | Line numbers 134-136. |
| 27 | In 2019, the population of CLMV countries was 174 million and this is approximately 25% of the population of Association of South East Asia Nations (ASEAN) members and 3.6% of the total population of Asia: there is no connection between this sentence and the previous one. provide a reference here. | The authors removed these sentences. |  |
| 28 | Gross domestic product (GDP) per capita in 2019 averaged 2,200 US$, much lower than the ASEAN average of 12,979 US$ and the Asian average of 17,909 US$: The authors should remove the abbreviation GDP as it is not well known or international. The authors also should think about the necessary of this sentence. Also to make sure that all sentences are well connected to each other. | The authors revised the sentences such as " Consequently, in 2019, Gross Domestic Product per capita of CLMVs averaged 2,200 US$, still much lower than the ASEAN average of 12,979 US$ and the Asian average of 17,909 US$, showing less development [8]. | Line numbers 136-138. |
| 28 | The CLMV countries managed to reduce the maternal mortality rate by 32% to reach the target of a 2/3 reduction in MMR over the years (from 1990 to 2015): a reference is needed here. | The authors added reference number 2 to the sentences, "In ASEAN, the CLMV countries have the highest maternal mortality rate in comparison with other LMICs. The CLMV countries managed to reduce the maternal mortality rate by 32% to reach the target of a 2/3 reduction in MMR over 25 years (from 1990 to 2015) [2]." | Line number -141. |
| 29 | In the CLMV countries, the average MMR of Cambodia from 1990 to 2020 was 276/100,000 LB and the measure in 2020 indicates that 206/100,000 LB need to be reduced for achieving the SDG target by 2030 (<70/100,000 LB). The average MMR of Lao PDR from 1990 to 2020 was 362/100,000 LB and the 2020 MMR data indicates that 292/100,000 LB need to be dropped between 2021 and 2030. The average MMR of Myanmar from 1990 to 2020 was 186 and the 2020 MMR data pointed out that it needs to drop more than 116/100,000 LB to meet the SDG target (less than 70/100,000 LB). When looking at Vietnam, the average MMR from 1990 to 2020 was 199/100,000 LB: references are needed here. Make good connection to these sentences to make them more readable. | The authors rewrote these sentences " Particularly, as the average MMR of Cambodia from 1990 to 2020 was 276/100,000 LB, the measure in 2020 still indicates that 206/100,000 LB need to be reduced for achieving the SDG target by 2030 (<70/100,000 LB). For Lao PDR, its average MMR from 1990 to 2020 was 362/100,000 LB and its 2020 MMR data indicated that 292/100,000 LB need to be dropped between 2021 and 2030. In Myanmar, its average MMR from 1990 to 2020 was 186 and its 2020 MMR data pointed out that it needs to drop more than 116/100,000 LB to meet the SDG target (less than 70/100,000 LB). When looking at Vietnam, the average MMR from 1990 to 2020 was 199/100,000 LB. Also, Vietnam needs to remove more than 129/100,000 LB from the 2020 MMR figure to achieve the SDG target (less than 70/100,000 LB) [2]." | Line numbers 142-150. |
| 30 | The CLMV countries managed to reduce the maternal mortality rate by 32% to reach the target of a 2/3 reduction in MMR over the years (from 1990 to 2015): a reference is needed here. | As per the reviewer's comment, the authors added a page number. " The maternal mortality trends among CLMV countries (1990-2020) were shown in S2 Fig (Page number (7))." | Line numbers -150-152 |
| In the sixth paragraph, | | | |
| 31 | When estimating the average annual MMR (per 100,000 LB) of reduction, 7.1 could be reduced in Cambodia, 14.9 in Laos, 0.4 in Myanmar and 3 in Vietnam: please write the reference here.  In these cases, among CLMV  countries, there are still huge differences between the average annual MMR of reduction and the annual MMR needed to achieve the SDG target: reference is needed here. | The authors have already added reference number (2) such as " When estimating the average annual MMR (per 100,000 LB) of reduction, 7.1 could be reduced in Cambodia, 14.9 in Laos, 0.4 in Myanmar and 3 in Vietnam. Actually, for achieving the SDG target of 70 maternal deaths (MD)/100,000 LB in 2030, Cambodia needs to reduce 20.6 MD/100,000 LB, Laos needs to reduce 36.2 MD/100,000 LB, Myanmar needs to drop 11.6 MD/100,000 LB and Vietnam need to reduce 12.9 MD/100,000 LB annually. In these cases, among CLMV countries, there are still huge differences between the average annual MMR of reduction and the annual MMR needed to achieve the SDG target [2]. | Line numbers 153-160. |
| Overall | | | |
| 32 | The authors need to arrange the introduction section. Rewrite some sentences so, the readers will understand what you wanted to convey from messages. The authors should make sure that missing references are written within the introduction section. They also need to make sure to clearly mention the significance of the systematic review and the aim of it. The authors should indicate what they wanted the readers to understand. The authors should indicate the significance of the systematic review and the overall aim of this systematic review. | The authors have revised and re-arranged the introduction section of the manuscript as per reviewer's comments. | Line numbers -60-166. |
| **Material and Methods:** | | | |
| Research Design | | | |
| 33 | The authors should write one sentence on how to limit the bias for this systematic review. | In this study, the researchers wanted to summarize all possible factors influencing the improvement of MMR in CLMV countries by limiting avoidable biases such as paper selection bias, data extraction bias, analysis and interpretation bias and confounding bias. | Line numbers 170-172. |
| 34 | (available at <https://prisma.shinyapps.io/checklist/>): no need to mention this in the paper, however, the authors can mention or refer to this link in the appendix. | The authors removed the link. | Line number 177. |
| 35 | The research question of the review was "Which factors are influencing the improvement of maternal deaths in Cambodia, Laos, Myanmar, and Vietnam, CLMV countries?": Please, rewrite the question in a better way. For example, what are the factors affecting……? | The authors re-wrote the research question such as "The research question of the review was "What are the factors influencing the improvement of maternal deaths in Cambodia, Laos, Myanmar, and Vietnam, CLMV countries?"" | Line numbers 177-179. |
| 36 | (see in S3 Fig): the authors should mention the page number, so the reader can access to information better and fast. | As per the reviewer's comment, the author added page number to this sentence "To construct the question, the researcher applied the "PICOT" criteria (see in S3 Fig) (page-8)." | Line number 179-180. |
| 37 | According to the argumentation of a study, the appropriateness and specificity …..: Delete according to the argumentation. | As per the reviewer's comment, the authors deleted the phrase such as "The appropriateness and specificity of the research question were checked with the FINER (Feasible, Interesting, Novel, Ethical, Relevant) criteria for being a good research question [9]" | Line numbers 180-182. |
| 38 | In Table 1. Research question and FINER criteria: in Feasible (F), why did you exclude purchased papers or studies? Provide reasons. in Interesting (I), you wrote (Found more effective ways to reduce MMR to the referenced targets of the SDG goals) but your aim is to know the factor, please check that and if you have more than one aim, please write them. | As per the reviewer's claim, the authors explained the reasons that "Exclude the purchased papers because this dissertation work was done by student's fund." And the authors removed the sentence " Found more effective ways to reduce MMR to the referenced targets of the SDG goals" | Line number 184 (in table 1) |
| 39 | The authors should mention if a protocol is exist for their systematic review. | The authors have already mentioned the review protocol in the section on ethical considerations such as " Before commencing this review, the researchers submitted an ethical form and review protocol to both institutional review boards of STIMU, Myanmar and the University of Bedfordshire, the UK for approval. Both boards approved the protocol on 13^th^ July 2022 with the SSCHREC application number (STI011-6-2022-3-003)." | Line numbers -347-351 |
| **Inclusion/ exclusion criteria** | | | |
| 40 | The elements of PICOT that were mentioned in S3 Fig addressed the inclusion criteria of this review: please write page number for S3. | As per the reviewer's comment, the author added page number to this sentence "To construct the question, the researcher applied the "PICOT" criteria (see in S3 Fig) (page-8)." | Line number 179-180. |
| 41 | The authors should write clearly PICOT stand for what, so the readers will understand. | The authors explained the stands of PICOT such as " The elements of PICOT (P = Population studies, I = Intervention, C = Comparison, O = Outcome and T = Time Frame) that were mentioned in S3 Fig addressed the inclusion criteria of this review." | Line numbers 189-191 |
| 42 | The authors should separate the paragraphs of inclusion criteria and exclusion criteria, so the reader will follow the systemic review in more clear way. | The authors re-arranged the inclusion and exclusion criteria such as " ***Inclusion criteria***  The criteria of the papers to be included in the review were totally based on the basic structure of the review question. The elements of PICOT (P = Population studies, I = Intervention, C = Comparison, O = Outcome and T = Time Frame) that were mentioned in S3 Fig addressed the inclusion criteria of this review.  This systematic review typically focused on primary studies conducted using a specific type of qualitative and quantitative approach or a type of mixed qualitative and quantitative technique. The justification for the selection of only primary research was that the researcher wanted to collect more specific and conclusive information about the influencing factors of MMR. Besides, it was concluded that a methodology of mixed-methods study can also generate a convergence of more exact quantitative evidence and the behaviours, feelings, and attitudes of the targeted population [10]. Here, the researcher wanted to overcome the weaknesses of an approach with the strengths of another approach and vice versa in summarizing the evidence regarding the determinant factors of maternal death. This was a reason why the researcher primarily concentrated on a mixed-methods approach.  The choice of studies, which were published between 2010 and 2021, inspired the researcher's interest in the subject. The 11-year evaluation period was not excessively long and could deliver essential, accurate, and appropriate data on the maternal mortality situation in CLMV countries. It was discussed that a long evaluation period of a systematic review might conclude the outdated issues that had already been addressed whereas a very short time bound of its evaluation might generate incomplete evidence [11]. The researcher wanted to explore the associated factors of MMR trends before and after the targeted date of MDGs (2015) as well as during the ongoing period of SDGs. The interesting point was that long-duration due to the global policy changes in 2015 from MDGs to SDGs. The MDGs were scheduled to conclude in 2015, and the argument over a post-2015 agenda is still ongoing.  This review was more likely to choose the studies conducted in four low-income countries of Asia: Cambodia, Laos, Myanmar, and Vietnam as the priority. The main rationale for selecting research that studied CLMV countries was that the researcher wanted to assemble all possible influencing factors on the slow declination of MMR and the reasons for being behind the global targeted settings within these countries.  The key study population for this systematic review was the pregnant, childbirth, post-natal women and the reproductive population at community levels from CLMV countries, followed by the healthcare professionals who were primarily in charge of providing maternity and primary healthcare services in different healthcare settings. To obtain accurate results and thorough information on these factors, the study considered the male partners and the authorized healthcare persons operating in the national health system to be included. Details of this systematic review's primary exposures of interest and reported outcomes have already been mentioned with the "PICOT" framework (S2 Fig).  ***Exclusion criteria***  This review excluded secondary analysis and evidence-based information that has previously been published as historical documents, systematic reviews or/and meta-analyses, reports, books, handouts, posters, factsheets, encyclopedias, student thesis, and conference proceedings. The review also excluded others that did not agree with the inclusion criteria." | Line numbers 189-230 |
| 43 | This review excluded secondary analysis and evidence-based information that has previously been published as historical documents, systematic reviews or/and meta-analyses, reports, books, handouts, posters, factsheets, encyclopedias, student thesis, and conference proceedings: The authors should provide reasons for exclusion briefly and what the authors means when they wrote exclude secondary analysis that has previously been published. | The authors added the reasons for exclusions such as "The reason for these exclusions was that the reviewers wanted to analyze the outcome information from the primary research efforts focusing on specific research question with detailed research methodology. The review also excluded others that did not agree with the inclusion criteria." | Line numbers 230-233. |
| 44 | "PICOT" framework (S3 Fig). Please write the page number of S3 figure. | The authors added the page number following S3 Fig. such as " Details of this systematic review's primary exposures of interest and reported outcomes have already been mentioned with the "PICOT" framework (S3 Fig) (page-8)." | Line numbers 222-224 |
| **Selection of Information Sources** | | | |
| 45 | …s, etc.,: please, no need the write extra. Just write what you searched. Or delete this sentence since you used electronic resources only. | As per the reviewer's comment, the authors re-wrote this sentence " In this review, the researcher used only electronic resources, for which the Internet was essential." | Line numbers 241-242. |
| 46 | Table 2. List of the Selected Academic Research Databases and their Descriptions: the authors can put this table in appendix and then refer it with page number. | The authors put this table according to the journal's requirement. |  |
| 47 | The authors can write the number of study from these three database briefly. | The authors mentioned the number of studies from the three databases in Figure (1)- Flow diagram of the paper selection process. | Figure (1). |
| **Defining Key Search Terms** | | | |
| 48 | "Influencing Factors", "Maternal Mortality and Health Outcome": I think these keywords should be separated, for example, no need to mention influencing factors (factor is enough), maternal is one keyword and mortality is another keyword. Please check that. | As per the reviewer's comment, the authors re-defined the keywords " the researcher used four key phrases: "Factors", "Maternal Mortality and Health Outcome", "Maternal Healthcare Interventions" and "CLMV Countries"." | Line numbers 255-257.  Table 3. |
| **Search Method (Organizing review team)** | | | |
| 49 | coordinator of STIMU: please, write the full form of STIMU. | The authors wrote the full form of STIMU such as "Science & Technology International Myanmar University (STIMU)" | Line number -268. |
| 50 | on 13 July 2022, 28 July 2022 and 11 August 2022 and stopped in all databases on 1st September 2022: I think you need to mention the date from …to…. For example, for 13 July 2022 to 1^st^ Sep 2022. | As recommended by the reviewers, the authors corrected the sentence "The independent literature search was completed in Hinari, Google Scholar and PubMed from 13 July 2022 to 1^st^ September 2022." | Line numbers 271-272. |
| Developing tools | | | |
| 51 | The researcher: The authors have to be consistent using the word the researcher or the researchers or investigators in all document.  The authors should check grammar for readability. | The authors corrected the term "investigator" with the use of the typical term "researcher". | Line number -243  Line number -254  Line numbers 265-266  Line number -279  Line numbers -282-288  Line numbers -301-303  Line number -310.  Line number -316 |
| Study Selection | | | |
| 52 | Details of selection processes were explained in the finding section: please refer to the page number, so the readers can get access fast.  PRISMA: the authors can provide diagram of the PRISMA in this systematic review. | The authors mentioned the page number following the sentence " Details of selection processes were explained in the finding section (page 22)." | Line numbers -311-312. |
| Information Extraction | | | |
| 53 | DET: the authors have to write the full form of this abbreviation. | The authors mentioned the full form of DET such as "data extraction template (DET)" | Line number -316. |
| Quality Appraisal | | | |
| 54 | The authors should provide the reason for selecting JBI.  The authors should check grammar for readability. | The authors explained the reason for JBI use such as "The researchers' reason for JBI use was that the appraisal tool set was capable of assessing the methodological qualities of the different study designs and trustworthiness and relevancy of research outputs." | Line numbers 332-334. |
| Data Analysis | | | |
| 55 | In qualitative analysis, among different approaches: content analysis, narrative analysis, discourse analysis, framework analysis and grounded theory, this review analyzed all key extracted information through content analysis: the authors should provide reasons for selecting these data analysis. It is well known in grounded theory that constant comparative analysis.  Finally, the highlighted major themes were summarized and assembled according to the representatives of eight themes: Please write these 8 themes. | The authors corrected these sentences such as " The reason for applying content analysis, the analysis help researcher to identify themes, patterns and relationships of the key extracted information, apply a manual method to exercise the open and axial coding of similar findings and categorize eight themes (socio-economic factors, maternal biological factor, maternal health awareness-associated factor, maternal health need-associated factor, maternal psychological factor, status and quality of the maternal healthcare workforce, maternal healthcare service use-associated factor and community-associated factor) as predefined in the review objective setting." | Line numbers -346-352. |
| Ethical consideration | | | |
| 56 | STIMU and SSCHREC: Please write the full form of these two abbreviations.  Overall, the authors should revise the language and grammar to improve readability. I think, the authors should arrange this section in a better way. Please refer to the guideline of this journal. | The authors rewrote these sentences "This systematic review has very few ethical concerns because the researchers did not directly collect any personal, sensitive and confidential information of human subjects [13]. Besides, before commencing this review, the researchers submitted the review protocol with an ethical form to both institutional review boards of Science & Technology International Myanmar University (STIMU), Myanmar and the University of Bedfordshire, the UK for approval. Both boards approved the protocol on 13^th^ July 2022 with the Ethics Committee of the School Society Community and Health (Public Health) (SSCHREC) application number (STI011-6-2022-3-003). During the review, the researcher used only publicly accessible documents and abstained from abnormal minimization or maximization of the original research findings." | Line numbers -357-365. |
| Results  Summary of Paper Selection Process | | | |
| 57 | The authors should move this section to the PRISMA. | The authors would like to request to place this section there as per the PRISMA checklist serial arrangement. |  |
| 58 | Two investigators first identified 1112 titles and abstracts by searching PubMed (31.3%), Google Scholar (44.6%), and Hinari (24.1%) records: the authors should mention the number of studies identified in each database, I don’t understand what is the meaning of the percentages of each database. | The authors mentioned the number of studies from the three databases in Figure (1)- Flow diagram of the paper selection process.  As per the reviewer's comments, the authors deleted the percentages such as " Two researchers first identified 1112 titles and abstracts by searching PubMed (n=348), Google Scholar (n=496), and Hinari (n=268) records." | Figure (1).  Line numbers -369-370. |
| 59 | Inter-reviewer agreement form: the authors should explain the meaning of this form briefly. Then, the authors should mention how many papers excluded and why. | The authors have already explained the details of "Inter-reviewer agreement form" under the sub-heading "Developing Tools" in the Material and Method section (Page .19)  The authors have mentioned the number of papers excluded and reason for exclusions in S1. Fig. (PRISMA Flow Diagram) | Line number -282-288.  S1. Fig. |
| 60 | see in S1 Fig: mention the page number please after fig. | The author mentioned the page number after S1. Fig such as " Finally, 75 (6.7% of total identified records) full-text papers remained for qualitative analysis (see in S1 Fig) (page. 23)." | Line number -376. |
| Background information if included studies | | | |
| 61 | Since this study concentrated on the CLMV countries, all included studies took place in these four countries. 27 (36%) of the 75 studies were from Myanmar, 23 (31%) from Laos, 13 (17%) from Cambodia, and 12 (16%) from Vietnam: I don’t think there is a need to mention percentages after each number, check that please for all document. | The author mentioned all frequency with numbers such as " Since this study concentrated on the CLMV countries, all included studies took place in these four countries. 27 of the 75 studies were from Myanmar, 23 from Laos, 13 from Cambodia, and 12 from Vietnam.  Among the 75 studies, 54 discussed maternal death-related socioeconomic factors, 42 discussed maternal health needs, 30 discussed the influencing factors of maternal health literacy promotion, 24 discussed the status and quality of maternal healthcare workforce, and 23 discussed health system and maternal healthcare services. 13 disclosed maternal death-related maternal biological factors, while the remaining 19 disclosed community-associated and maternal psychological factors influencing maternal health.  In terms of research designs, 36 studies were cross-sectional studies, 18 were qualitative research designs, 11 were secondary data analysis, 7 were cohort research designs, 2 were case-control research designs, and 1 was survey methods.  In this review, 19 studies were published between 2010 and 2014, and 25 between 2015 and 2018 and 31 between 2019 and 2022. And the total sample size for this systematic review was 2,361,463, with a sample size ranging from 23 to 2,214,339.  The quantitative information in this review was gathered using semi-structured interview questionnaires (n=62) and the qualitative information was gathered through focus group discussions and key informant interviews (n=13).  In terms of data analysis tools, 32 used multivariate analysis, 20 used descriptive analysis, 13 used thematic coding analysis, and the remaining 10 used logistic regression, operational analysis, sequential regression, and the student t-test." | Line numbers -379-399. |
| 62 | In this review, 31 (41.3%) of the papers were published between 2019 and 2022, 19 (25.3%) between 2010 and 2014, and 25 (33.33%) between 2015 and 2018: the authors should arrange papers according to the years, 2015 before 2014. | The authors re-arranged these sentences such as " In this review, 19 studies were published between 2010 and 2014, and 25 between 2015 and 2018 and 31 between 2019 and 2022." | Line numbers -391-392. |
| 63 | The quantitative information in this review was gathered using semi-structured interview questionnaires (83%) and the qualitative information was gathered through focus group discussions and key informant interviews (17%): the authors need to mention number of papers. Be consistence in all document, I think number if papers is better than percentage. | The author mentioned all frequency with numbers such as " Since this study concentrated on the CLMV countries, all included studies took place in these four countries. 27 of the 75 studies were from Myanmar, 23 from Laos, 13 from Cambodia, and 12 from Vietnam.  Among the 75 studies, 54 discussed maternal death-related socioeconomic factors, 42 discussed maternal health needs, 30 discussed the influencing factors of maternal health literacy promotion, 24 discussed the status and quality of maternal healthcare workforce, and 23 discussed health system and maternal healthcare services. 13 disclosed maternal death-related maternal biological factors, while the remaining 19 disclosed community-associated and maternal psychological factors influencing maternal health.  In terms of research designs, 36 studies were cross-sectional studies, 18 were qualitative research designs, 11 were secondary data analysis, 7 were cohort research designs, 2 were case-control research designs, and 1 was survey methods.  In this review, 19 studies were published between 2010 and 2014, and 25 between 2015 and 2018 and 31 between 2019 and 2022. And the total sample size for this systematic review was 2,361,463, with a sample size ranging from 23 to 2,214,339.  The quantitative information in this review was gathered using semi-structured interview questionnaires (n=62) and the qualitative information was gathered through focus group discussions and key informant interviews (n=13).  In terms of data analysis tools, 32 used multivariate analysis, 20 used descriptive analysis, 13 used thematic coding analysis, and the remaining 10 used logistic regression, operational analysis, sequential regression, and the student t-test." | Line numbers -379-399. |
| 64 | According to the JBI criteria, some cross-sectional studies (12.5%, n=6) did not mention pre- testing data collection instruments and Cronbach alpha values as well as the standardization of the instruments. And (25%, n=12) studies also did not explain how they identified the confounding factors: did these affected the review in some way, please explain briefly. | The authors mentioned the results of JBI appraisal work in the review report and the results had no any effects on inclusion and exclusion of the studies and extraction of outcomes. |  |
| Factors Influencing Maternal Death  Socioeconomic Factors | | | |
| 65 | The authors should join some paragraphs together which talk about the same concept. | The authors arranged the paragraphs according to the same concepts such as " *Socio-economic factors*  Women with lower socioeconomic status had less access to quality maternal and reproductive healthcare services and were at a higher risk of maternal death (Table 6). According to the findings of this review, family income was a significant factor that was strongly associated with maternal and reproductive healthcare access and maternal death outcomes [14, 15]. It was also found that poorer women were about 5 times (95% CI, 2.2-9.6) more likely than richer women to object to high-cost antenatal care services, obstetric and gynecological care, and ultrasound machine management [16-22].  Furthermore, occupation of both women and husbands was important for positive maternal health outcomes. Pregnancies were 40-45% less likely to be delivered in a hospital if their husbands worked in agriculture or unskilled manual labor [23] and the chances of developing post-partum complications were about 30% if the pregnancies were manual or agricultural laborers, The repetitive abortion rates (30-40%) among female sex workers were 20-26% higher compared to these rates (10-14%) among farmers [24].  It was also discovered that maternal health care services utilization and poor maternal health outcomes were highly correlated with mother education [25-27]. Furthermore, a study found that pregnant women with higher education received more than 7 times of antenatal care, while women with middle education levels received approximately 6 times of antenatal care, women with primary education levels received approximately 5 times of antenatal care, and those with no education received less than 4 times of antenatal care (P0.000) [28].  In terms of male involvement, having a supportive husband was positively correlated with current contraceptive use, antenatal care practices, and delivery plans. Women who had husbands who supported contraception were more likely to succeed in family planning than other women [29]. Similarly, the studies concluded that favorable decisions made by husbands, gender, and societal norms had a significant impact on women's access to antenatal care, contraceptive choice, and delivery options [30, 31]. However, it was discovered that most husbands' perspectives were that discussions about pregnancy care, family planning, and delivery cases were not their concerns [32].  In terms of residence, rural women (54.6%) were more likely than urban women (61.7%) to have lower institutional delivery and skilled birth attendance, indicating that geographical barriers influenced the availability of maternal healthcare services and maternal health outcomes (P < 0.01) [23, 32]. Long geographical distances and difficult-to-reach areas were major factors in the second delay of maternal deaths, and it was determined that the second delay was responsible for more than 80% of rural mother deaths [26].  Furthermore, rather than financial constraints, the delay in seeking care or the first delay that could lead to the mother's death was heavily influenced by certain social and cultural beliefs. Pregnant women's lack of empowerment, the influence of spiritual healers, a close relationship with traditional birth attendance, and medication misuse were all important factors in the delay in seeking care [18, 29, 33-35]. It was also discovered a culture in some ethnic groups that may lead to maternal death, in which pregnant women had to go into a forest for their births, where births were performed by themselves or with the assistance of their husbands, and then spend three days afterwards [21]. " | Line numbers -408-449 |
| 66 | In terms of male involvement, having a supportive husband was positively correlated with current contraceptive use, antenatal care practices, and delivery plans. Women who had  husbands who supported contraception were more likely to succeed in family planning than  other women [29]. Similarly, the studies concluded that favorable decisions made by husbands, gender, and societal norms had a significant impact on women's access to antenatal care, contraceptive choice, and delivery options [30, 31]. However, it was discovered that most husbands' perspectives were that discussions about pregnancy care, family planning, and delivery cases were not their concerns [32]: The author should check if husband support will be under the concept of socio-economic factors. | According to the authors' predefined eight themes, husband-related activities should be under the theme of socioeconomic factors. | Line numbers -428-435. |
| 67 | The authors should check that all the references in Table 6. The influence of socioeconomic factors on accessing maternal health care services and high risk of maternal deaths are correlated with the text. | As per journal requirement, or not to be duplicated information, the authors placed some information in text and some key information in Table 6. | Table 6. |
| Maternal biological factors | | | |
| 68 | Early and older marriage ages were found to increase the likelihood of not receiving quality antenatal care, and elderly pregnant women were more likely to rely on traditional therapies. Early childbirth age was statistically associated with birth-related complications and missed postnatal follow-up care (Table 7) [22, 34]: the authors should mention if marriage age affect MMR within these studies. | The authors have already discussed the effect of marriage age on maternal death in the discussion section.  " Early and older ages at marriage were shown to increase the risk of inadequate prenatal care, and older pregnant women were found to be more likely to rely on traditional remedies." | Line numbers -617-619. |
| **Maternal Health Awareness-Associated Factors** | | | |
| 69 | According to one study, approximately one-third of HIV-positive pregnant women had less access to maternal health education and family planning education [48]: please mention the result of this study if MMR increased. | The paper [48] mentioned that approximately one-third of HIV-positive pregnant women had less access to maternal health education and family planning education and did not mention MMR among the study population. |  |
| 70 | Furthermore, young women and primigravidae had significantly lower rates of sexual reproductive health discussion [47] and rural mothers had a lower awareness of pregnancy and childbirth complications, as well as postpartum dietary restrictions [49, 50]: again, the authors need to discuss if the concept of these studies like health education or awareness had affect MMR negatively or positively. | The authors explained the effects of this concept on MMR in term of antenatal care coverage, skill birth attendance, and institutional births indirectly in Table 7. | Table 7. |
| **Maternal Health Need-Associated Factor** | | | |
| 71 | The authors need to separate the table of this factor from the next factor. | The authors separated Table 7 into Table 7, 8 and 9. | Table 7,8, and 9 |
| **Maternal Psychological Factors** | | | |
| 72 | The authors need to separate the table of this factor from the previous factor | The authors separated Table 7 into Table 7, 8 and 9. | Table 7,8, and 9 |
| **The Status and Quality of The Maternal Healthcare Workforce** | | | |
| 73 | It was also claimed that TBA handled pregnancy and childbirth complications in an unscientific and unsafe manner, which could result in maternal deaths [18]: The authors should clarify and mention what is the meaning of TBA abbreviation. ABA Stand for what? | The authors clarified TBA such as "the trained birth attendance (TBA) handled pregnancy and childbirth complications in an unscientific and unsafe manner, which could result in maternal deaths" | Line numbers -510-511. |
|  | The authors should revise the grammar for more readability. | The authors revised these sentences such as " Relating to the quality of the maternal healthcare workforce, it was claimed that most maternal deaths came from home deliveries with untrained traditional birth attendances [14, 34]. It was also claimed that the trained birth attendance (TBA) handled pregnancy and childbirth complications in an unscientific and unsafe manner, which could result in maternal deaths [18]. Maternal deaths were found to be affected by the entry of new healthcare providers (e.g., fresher Mid-wives with less expertise on childbirth and revision of maternal complications), the lack of experience during births (e.g., less one year of service), the lack of continuous medical education, and the low quality of basic healthcare providers [28, 33, 60, 61]." | Line numbers -508-515. |
| 74 | Furthermore, underdevelopment of healthcare infrastructures and a lack of medical supplies were identified as major barriers to providing effective care and high-quality services [21, 62]: I think this should come under the next factor as it discuss the services. | The authors moved these sentences to next section (Maternal Healthcare Service Use-associated Factors). | Line numbers -529-531. |
| **Maternal Healthcare Services Use-Associated Factors** | | | |
| 75 | The authors should revise the grammar for more readability. | The authors revised the paragraph such as " In CLMV countries, women from some areas were restricted from accessing and using reproductive healthcare facilities, affecting the regularity and quality of antenatal care services and contributing to high maternal mortality ratios. According to one study, the frequency of mothers receiving antenatal care services was very low in hilly, remote, and rural areas although mobile and field maternal healthcare services were provided for higher coverage of antenatal care [15]. Furthermore, many complaints of local pregnant women were reported, including low satisfaction with many supply-side issues, payments for public maternal healthcare services, under-quality services in health facilities, very long waiting times, negligence of patient complaints, and less dutiful responsibilities [18]. Additionally, underdevelopment of healthcare infrastructures and a lack of medical supplies were identified as major barriers to providing effective care and high-quality services [21, 62]." | Line numbers -521-531. |
| **Community-Associated Factors** | | | |
| 76 | The authors need to discuss how the community associated factors affected MMR. | The authors discussed the relationships between the importance of community-associated factors and access to maternal healthcare services that directly effect on maternal deaths. | Line numbers -545-555. |
| 77 | The authors should revise the grammar for more readability. | The authors rewrote this paragraph such as " The main sources of uncertainty in maternal and reproductive healthcare coverage were a lack of community empowerment and a lack of training for volunteer health workers (VHWs), particularly in hard-to-reach areas [15]. The VHWs could help with the requirements for timely referral (e.g., vehicles, carriers, and manpower), overcoming language barriers, and reducing second delay that is one main cause of maternal death. However, for effective maternal healthcare, community involvement and the availability of VHWs could not overcome some barriers such as long distances and walking time to the nearest medical centers [14, 30, 32, 33]. On the other hand, village leadership and collaboration with healthcare professionals had a greater impact on community-based maternal healthcare services [21]. Furthermore, it was critical for healthcare providers to have mutual respect and effective communication with community leaders in order to improve the quality and demand for maternal healthcare services [51]." | Line numbers -545-556. |
| Discussion | | | |
| 78 | In this study, family income was shown to be a strong and significant factor that was substantially related to maternal and reproductive healthcare access and maternal mortality: mention review instead of study and mention the references discuss this concept. | The authors changed this sentence such ae " In this review, family income was shown to be a strong and significant factor that was substantially related to maternal and reproductive healthcare access and maternal mortality [64]." | Line numbers -559-561. |
| 79 | The authors discussed the all factors but need to discuss from different angles. They should discuss clearly about the issues found from different angels.  The authors should discuss each themes from different angle and in detail  The authors should revise the language and grammar to improve readability.  Most of discussion section was recommendation, the authors should revised this section.  The authors should discuss the results in consistent with the previous section.  Overall, Discussion section should be revised totally and should be discussed from different angels | The author revised some points such as "Actually, the governments of CLMV countries are less ability to propose the initiative programs such as the cost share program for maternal health care services and a pregnancy-peer group fundraising programme. Moreover, the stakeholders in CLMV countries have small numbers of the local health committee and pregnancy and baby love organizations which can pool the necessary funds and support other necessities for poor pregnant women [6]."  In this issue, the authorities of the Department of Health from CLMV countries insufficiently practice media roles such as (Television, Radio, Facebook, and Channel Programmes) rather than Vinyl, posters, pamphlets, books, journals, magazines and newspapers in maternal health awareness promotion campaigns among illiterate pregnant women. Further, sex education programmes in schools could not continuously be expanded and implemented with the cooperation of the Ministry of Health and the Ministry of Education to distribute maternal and reproductive health messages from students to their families to their communities [6].  In spite of this importance, the authorities from CLMV countries stills require widely implement intensive public education on the importance of male involvement in the Safe Motherhood Programme and couple counselling practices to promote the roles of the husbands in antenatal care services and during the delivery [6].  Especially, in CLMV countries, there are a smaller number of metropolitan areas that typically have better emergency obstetrics services than rural [6]. Women in rural regions may not be aware of the significance of the conditions that are linked to maternal mortality due to inadequate health facility resources.  Teenage mothers and women who are towards the end of their reproductive years are shown to be at the highest risk of maternal death. Maternal death rates are significantly higher in early marriage and teenage pregnancies (under 16 years old). This may be because young women's pelvic structures are not ready to carry fetuses, which increases the chance of labour obstruction and, in the worst-case scenario, may result in maternal death. In addition, early marriages subject young women to early parenthood and complicated pregnancies that could result in maternal mortality. Because of their physiologic immaturity and general lack of social and economic resources, adolescent females are known to experience several obstetric problems more frequently and with greater severity. These include perinatal mortality, preterm birth, low birth weight, premature birth, anaemia, malnutrition, cephalopelvic disproportion, vesicovaginal and recto-vaginal fistulae, and pregnancy-induced hypertension.  The greatest number of maternal deaths occurred in the age groups of less than 20 years and more than 35 years and among multiparous women. Ageing and a higher risk of pregnancy complications brought on by non-obstetric diseases are contributing factors in older pregnancies (those involving women over 40). Further, the number of pregnancies a woman has affects the risk that she would pass away during pregnancy or childbirth. Women who have their first child (Primigravida, P=0) or five or more children (Multigravida, P>4) are thought to have an increased risk of dying from maternal causes. Additionally, a close succession of pregnancies and lactation periods worsen the mother's nutritional status because there is insufficient time for the mother to recover from the physiological stresses of the preceding pregnancy before she is subject to the stresses of the next pregnancy. Consequently, it has been discovered that multigravidas had higher rates of maternal death than primigravida [68-70].  To cut the strong association between maternal age and maternal mortality, contraception is available and effective but the expansion of family planning healthcare centers and counselling services are less effective in CLMV countries [6].  It was discovered that literacy level and maternal fatalities were associated, which may be attributed to many effects of knowledge for healthy practice and health service consumption. Therefore, it's crucial to provide knowledge about reproductive health services and promote health to lower maternal mortality.  Understanding the factors that influence maternal mortality at the community level is essential from the standpoint of policy because this is the level at which most policy is implemented. The implementation gap may be evident when the lack of linkages between interventions in health facilities and the communities they serve occurs. Community participation is used as a strategy to understand these local realities, especially in the field of health promotion, and to include local communities in the planning and implementation of health programs including maternal and child health. Community participation creates an opportunity for community members to share challenges related to maternal and child health and ask for help [74]. | Line numbers -567-572.  Line numbers -580-587.  Line numbers -597-600  Line numbers -611-614.  Line numbers -619-630.  Line numbers -633-644.  Line numbers -644-647.  Line numbers -658-661.  Line numbers -672-680. |
| References | | | |
| 80 | The authors should revise all references according to the guidelines provided. | The authors revised all references according to the guidelines provided. | Line numbers -891-1219 |
| October, 3, 2023 | | | |
| 1 | Please review your reference list to ensure that it is complete and correct. If you have cited papers that have been retracted, please include the rationale for doing so in the manuscript text, or remove these references and replace them with relevant current references. Any changes to the reference list should be mentioned in the rebuttal letter that accompanies your revised manuscript. If you need to cite a retracted article, indicate the article’s retracted status in the References list and also include a citation and full reference for the retraction notice. | The reference " The Goalkeepers Report. Global progress and projections for maternal mortality. 2021. Available from: <https://www.gatesfoundation.org/goalkeepers/report/2021-report/progress-indicators/maternal-mortality/>" was changed by the updated reference "  The reference " World Health Organization (WHO). Time to response. A report on the global implementation of Maternal Death Surveillance and Response. Geneva: Switzerland. WHO Press, World Health Organization" was changed by the reference " World Health Organization (WHO). Time to response. A report on the global implementation of Maternal Death Surveillance and Response. Geneva: Switzerland. WHO Press, World Health Organization, 2016."  The reference " Edoardo A. and Alan P. (2014) "The Systematic Review: An Overview", *AJN, American Journal of Nursing*: 2016: 114(3); pp. 53-58 doi: 10.1097/01.NAJ.0000444496.24228.2c." was removed and substituted by the updated reference "The World Bank. East Asia and Pacific: Sustained growth, Momentum Slowing. (2023). Available from: <https://www.worldbank.org/en/news/press-release/2023/10/01/east-asia-and-pacific-sustained-growth-momentum-slowing>. (Accessed 3 October 2023)."  The reference " Cindy S., Zoe J. and Alexa M. "Developing the Review Question and Inclusion Criteria', AJN, *American Journal of Nursing*, 2024: 114(4) – pp. 53-56 doi: 10.1097/01.NAJ. 0000445689.67800.86" was removed and substituted by the updated reference " Stern C, Jordan Z, McArthur A. Developing the review question and inclusion criteria. *Am J Nurs*. 2014 Apr;114(4):53-6. doi: 10.1097/01.NAJ.0000445689.67800.86. PMID: 24681476."  The reference "Montesanti SR. 'Cultural perceptions of maternal illness among Khmer women in Krong Kep, Cambodia', vis-à-vis: *Explorations in Anthropology*, 2011: 11(1) (Accessed August 2022)" was changed by the reference " Montesanti SR. 'Cultural perceptions of maternal illness among Khmer women in Krong Kep, Cambodia', vis-à-vis: *Explorations in Anthropology*, 2011: 11(1), pp-90-106.  The reference "Smith LC. “The importance of Women's status for child nutrition in developing countries: international food policy research institute (IFPRI) research report” abstract 131. *Food and Nutrition Bulletin*, 2003: 24(3), pp.287-288." Was changed by the reference " Smith, L. C., Ramakrishnan, U., Ndiaye, A., Haddad, L., & Martorell, R. (2003). The importance of women’s status for child nutrition in developing countries: International Food Policy Research Institute (Ifpri) research report abstract 131. *Food and Nutrition Bulletin,* *24*(3), 287–288. | Line numbers –893-895  Line numbers -911-913.  Line numbers – 914-917.  Line numbers -927-929.  Line numbers -1014-1016  Line numbers -1081-1084. |
| 2 | The inclusion of the inclusion and exclusion criteria chart and deleting the repetitive parts of the discussion | The authors revised the statements " The criteria of the papers to be included in the review were totally based on the basic structure of the review question. The elements of PICOT (P = Population studies, I = Intervention, C = Comparison, O = Outcome and T = Time Frame) that were mentioned in S3 Fig addressed the inclusion criteria of this review" as follows.  " The criteria of the papers to be included in the review were totally based on the basic structure of the review question. The elements of PICOT (P = Population studies, I = Intervention, C = Comparison, O = Outcome and T = Time Frame) that were mentioned in S3 Fig (page.8) addressed the inclusion criteria of this review."  The authors deleted the statements " The key study population for this systematic review was the pregnant, childbirth, post-natal women and the reproductive population at community levels from CLMV countries, followed by the healthcare professionals who were primarily in charge of providing maternity and primary healthcare services in different healthcare settings. To obtain accurate results and thorough information on these factors, the study considered the male partners and the authorized healthcare persons operating in the national health system to be included. Details of this systematic review's primary exposures of interest and reported outcomes have already been mentioned with the "PICOT" framework (S3 Fig) (page-8)." | Line numbers -188-191.  Line numbers -217-224. |
| 3 | While revising your submission, please upload your figure files to the Preflight Analysis and Conversion Engine (PACE) digital diagnostic tool, <https://pacev2.apexcovantage.com/>. PACE helps ensure that figures meet PLOS requirements. To use PACE, you must first register as a user. Registration is free. Then, login and navigate to the UPLOAD tab, where you will find detailed instructions on how to use the tool. If you encounter any issues or have any questions when using PACE, please email PLOS at [figures@plos.org](mailto:figures@plos.org). Please note that Supporting Information files do not need this step. | The authors have already uploaded all figure files to the Preflight Analysis and Conversion Engine (PACE) digital diagnostic tool, <https://pacev2.apexcovantage.com/>. PACE and downloaded all figures.tif from PACE. |  |
